# Leveraging transcriptomic, DNA methylation, and molecular alteration data to optimize the classification of IDH-mutant gliomas for therapy selection

**DOI:** 10.3389/fonc.2025.1674987

**Published:** 2026-01-12

**Authors:** L. Nicolas Gonzalez Castro

**Affiliations:** 1 Center for Neuro-Oncology, Dana-Farber Cancer Institute, Boston, MA, United States; 2 Center for Tumors of the Nervous System, Mass General Brigham, Boston MA, United States; 3 Department of Neurology, Mass General Brigham, Boston, MA, United States; 4 Department of Pathology, Mass General Brigham, Boston, MA, United States; 5 Broad Institute of Harvard and MIT, Cambridge, MA, United States; 6 Harvard Medical School, Boston, MA, United States

**Keywords:** single-cell transcriptomic analysis, DNA methylation, WHO classification of central nervous system tumors, IDH inhibitors, IDH-mutant astrocytoma, IDH-mutant and 1p/19q co-deleted, IDH-mutant gliomas, oligodendroglioma

## Introduction

The 2021 World Health Organization (WHO) Classification of Central Nervous System (CNS) Tumors established isocitrate dehydrogenase (IDH) mutational status as a critical branching point in the classification of adult diffuse gliomas (1, 2). The discovery of IDH mutations in gliomas identified a subclass of tumors with a distinct epidemiology, biology and clinical behavior (3, 4). While glioblastoma, the most common and aggressive primary brain tumor, is often diagnosed in patients in their seventh or eighth decade, is driven by multiple genetic alterations, and has an invariably poor prognosis in the order of 16 months, IDH-mutant gliomas affect younger patients (mainly those in their 20s, 30s and 40s), are initially driven by epigenetic dysregulation (a result of the activity of the mutant IDH oncometabolite 2-hydroxyglurarate, 2-HG) and are characterized by a more effective response to therapy as well as a longer overall survival (4).

Radiation therapy remains the backbone of therapy for IDH-mutant gliomas, being offered to patients of all grades, including those with low-grade (grade 2) tumors that are older than 40 and had only biopsies or limited surgical resections (4). Although an effective treatment, radiation therapy is associated with long term cognitive decline, which can translate into significant impairment for IDH-mut glioma patients in their most productive years of their lives (5). In August 2024, the Food Drug Administration approved the oral dual mutant IDH1/2 inhibitor (IDHi) vorasidenib for the treatment of grade 2 IDH-mutant gliomas. The approval was based on the results of the phase 3 INDIGO study, that enrolled only grade 2 patients (6). In light of this new therapy, the question remains as to if patients with higher grade tumors are likely to benefit from IDHi therapy. This opinion piece calls into question the current relevance of the 2021 WHO classification of IDH-mut gliomas in light of this novel therapy, highlights recent developments that could lead to its update, and discusses the evidence in favor of expanding the use of IDHi therapy to patients with higher grade tumors.

## Current grading of IDH-mutant gliomas and impact on therapy selection

According to the 2021 WHO Classification of CNS tumors, IDH-mutant gliomas, can be classified as astrocytoma, IDH-mutant, WHO grade 2, 3, or 4, or oligodendroglioma, IDH-mutant, 1p/19q co-deleted, WHO grade 2 or 3 (1). Grade 2 tumors are characterized by mild to moderate cellular atypia without the presence of high-grade features such as necrosis or microvascular proliferation (1). Grade 4 tumors are characterized by marked atypia and prominently display elevated mitotic activity, necrosis and microvascular proliferation (1). In addition, homozygous deletion of tumor suppressor genes *CDKN2A* or *CDKN2B* classifies a tumor as grade 3 if oligodendroglioma, or grade 4 if astrocytoma, a sufficient but not necessary condition for these gradings. The classification of grade 3 tumors is more ambiguous, particularly for astrocytomas. Grade 3 tumors are required to have increased mitotic activity compared to grade 2 tumors, defined as greater or equal than 2 mitoses per high-power field, and no evidence of necrosis or microvascular proliferation (1). There are no molecular alterations that contribute to grading in grade 2 and grade 3 astrocytomas (1). According to data from the Central Brain Tumor Registry of the United States, grade 3 tumors account for 30.9% of IDH-mut astrocytomas and 32.6% of IDH-mut oligodendrogliomas (7). Per the current vorasidenib FDA approval label, these patients are not eligible for treatment with vorasidenib.

The question of whether patient with grade 3 IDH-mut glioma has not been addressed through a prospective clinical trial enrolling only these patients. However, review of the available literature on the use of IDH inhibitors in glioma patients reveals evidence of response in patients with grade 3 tumors. The initial study evaluating ivosidenib in IDH-mut glioma patients included patients with grade 3 tumors, and on the subset of these patients with non-enhancing disease, the median progression-free survival (mPFS) was 23 months, not different from the mPFS of 19.4 months observed in grade 2 non-enhancing tumors (8). A perioperative trial of vorasidenib and ivosidenib in patients with IDH-mut glioma, also enrolled patients with grade 3 tumors that demonstrated responses to treatment with both IDH inhibitors (9). More recently, a retrospective study on the use of ivosidenib in IDH-mut glioma also demonstrate partial responses and stable disease in patients with grade 3 tumors, with a mPFS and disease control rate that were not different from those observed for grade 2 tumors (10). Although vorasidenib has only been approved for grade 2 IDH-mutant gliomas, emergent evidence suggests that patients with grade 3 tumors are also likely to benefit from IDHi therapy, particularly those with non-enhancing tumors.

## Redefining IDH-mut glioma grading

The fact that the grading of IDH-mutant glioma according to the current WHO CNS tumor classification does not help predict what patients are likely to benefit from IDHi therapy compels us to consider approaches to refining the current classification. The clinical behavior of grade 2 and grade 4 tumors is well characterized, with the former demonstrating slow, often indolent, growth over many years, while the latter rapidly progressing over just a few years. As currently defined by the WHO classification, grade 3 tumors are thought to display intermediate behavior (11). However, it is unclear if this is the result of inherent grade 3 biology, or an averaging artifact from the behavior of upgraded grade 2 tumors and downgraded grade 4 tumors that are currently classified as grade 3. Emerging evidence suggests that there are only 2 grades for IDH-mut glioma – low-grade and high-grade – and that these gradings are not aligned with the current WHO gradings.

The application of single-cell methods to characterize the biology of cancer has helped advance our understanding of the diversity of transcriptional cellular states in a wide range of tumors (12). Cellular state heterogeneity is a hallmark of infiltrating gliomas (13), pioneering single-cell RNA sequencing (scRNA-seq) revealed that IDH-mut glioma display a cellular state architecture that mimics the normal developmental hierarchy of glioma, with malignant neural progenitor cell-like (NPC-like) cells giving rise to more differentiated – oligodendrocyte progenitor cell-like (OPC-like) and astrocyte-like (AC-like) – tumor cells (14, 15). A recent study profiling IDH-mut gliomas of different grades with single-cell multi-omic methods has revealed that grade progression is associated with expansion of the NPC-like compartment (16). These NPC-like cells are cycling cells that promote tumor growth and the study shows that size of the NPC-like cellular compartment is predictive of disease prognosis independent of grade (16). In fact, a recent study demonstrates that IDHi therapy promotes differentiation of proliferating NPC-like cells into more quiescent AC-like cells, providing a mechanism for the radiographic and clinical stability observed patients responding to treatment (17).

In addition, the expansion of the NPC-like cell compartment seen with grade progression in IDH-mut glioma is also accompanied by a progressive decrease in DNA methylation, so while low-grade IDH-mut gliomas are hypermethylated (as a result of the demethylating effect mediated by inhibition of demethylases by 2-HG), high-grade tumors are characterized by relative hypomethylation (16). This is in line with the methylome classification of IDH-mut gliomas, that separates IDH-mut astrocytomas and oligodendrogliomas into 2 distinct groups (18). Thus, if tumor cellular state composition could be characterized by scRNA-seq methods, or inferred via deconvolution of bulk-RNA-seq data (19), this information could be used to further refine IDH-mut glioma classification into novel low-grade and high-grade gradings that could better delineate patients likely to benefit from IDHi therapy. The classification could even be made more robust by incorporating DNA methylation data, which could help segregate tumors into one of these two groups.

Although RNA sequencing and DNA methylation analyses are not part of standard diagnostic workflows, current pathology workflows in place to characterize tumors according to the 2021 WHO classification already yield information that could help refine the classification of IDH-mut gliomas. As noted, *CDKN2A* and *CDKN2B* deletion status is currently assessed as evidence of homozygous deletion of either gene, establishes a grading of 3 for IDH-mut oligodendroglioma or a grading of 4 for IDH-mut astrocytoma. However, a recent study reviewing molecular tumor data from several institutions provided evidence that in IDH-mut astrocytoma, patients with grade 2 and 3 tumors with intact *CDKN2A* and *CDKN2B* status have comparable overall survival, while those with hemizygous loss or focal amplifications of *CDKN2A* and *CDKN2B* had a survival that was comparable to those with grade 4 tumors (20). These results provide evidence that readily available *CDKN2A* and *CDKN2B* alteration status data could be leveraged to classify IDH-mut astrocytomas into novel low-grade and high-grade gradings with distinct prognoses, which are also likely to discriminate between responders and non-responders to IDHi therapy.

## Discussion

**Figure 1:**
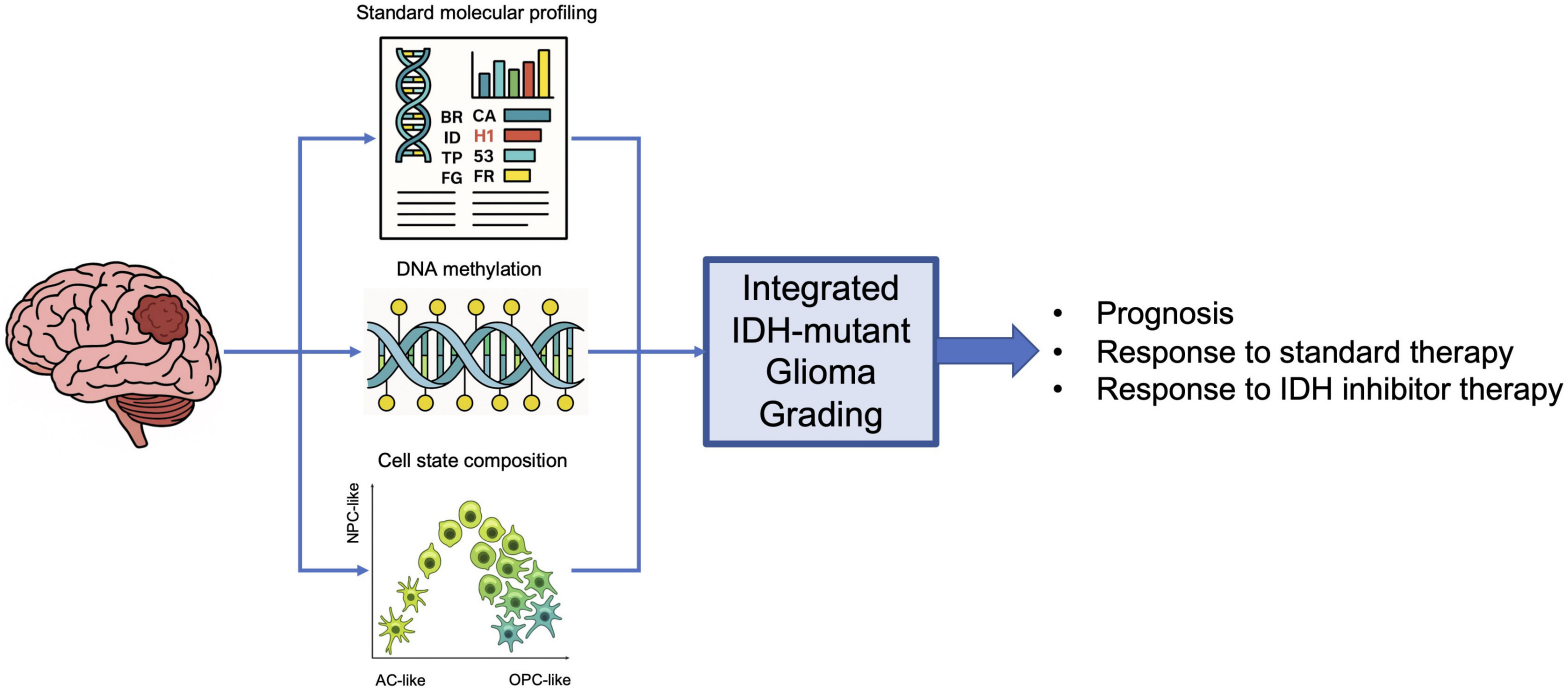
Integration of transcriptional cellular state, DNA methylation, and molecular alteration data to classify IDH-mutant gliomas and guide therapy selection.

The inclusion of molecular alterations in the criteria outlined in the 2021 WHO classification of CNS tumors has allowed us to more clearly dissect tumor types, leading to improvements in treatment and clinical trial selection, as well as more informed discussions in regard to prognosis. The results of the INDIGO study and the 2024 FDA approval of vorasidenib for the treatment of IDH-mut glioma, ushered a new era for the treatment of the most common brain tumors in young adults. Emerging data from studies evaluating IDH inhibitors in IDH-mut glioma indicate that patients with tumors grades other than 2 might also benefit from this therapy. Recent advances in characterizing changes in cellular state composition and DNA methylation in IDH-mut gliomas, as well as more detailed analyses of molecular alerations routinely evaluated to establish grade according to current criteria, could be leveraged to establish a more refined grading criteria, **Figure 1**. These criteria could more accurately identify all tumors likely to benefit from IDH therapy, while more readily singling out those that are not expected to respond and should instead be treated in the context of clinical trials or offered standard therapies.
